# Isopropanol production via the thermophilic bioconversion of sugars and syngas using metabolically engineered *Moorella thermoacetica*

**DOI:** 10.1186/s13068-024-02460-1

**Published:** 2024-01-28

**Authors:** Junya Kato, Takeshi Matsuo, Kaisei Takemura, Setsu Kato, Tatsuya Fujii, Keisuke Wada, Yusuke Nakamichi, Masahiro Watanabe, Yoshiteru Aoi, Tomotake Morita, Katsuji Murakami, Yutaka Nakashimada

**Affiliations:** 1 Graduate School of Integrated Sciences for Life, Hiroshima University, 1-3-1 Kagamiyama, Higashihiroshima, Hiroshima, 739-8530 Japan; 2 National Institute of Advanced Industrial Science and Technology (AIST), 3-11-32 Kagamiyama, Higashihiroshima, Hiroshima, 739-0046 Japan; 3 National Institute of Advanced Industrial Science and Technology (AIST), 1-1-1 Higashi, Tsukuba, Ibaraki 305-8565 Japan

**Keywords:** Isopropanol production, Biomass, Syngas, Thermophilic acetogen, Secondary alcohol dehydrogenase

## Abstract

**Background:**

Isopropanol (IPA) is a commodity chemical used as a solvent or raw material for polymeric products, such as plastics. Currently, IPA production depends largely on high-CO_2_-emission petrochemical methods that are not sustainable. Therefore, alternative low-CO_2_ emission methods are required. IPA bioproduction using biomass or waste gas is a promising method.

**Results:**

*Moorella thermoacetica*, a thermophilic acetogenic microorganism, was genetically engineered to produce IPA. A metabolic pathway related to acetone reduction was selected, and acetone conversion to IPA was achieved via the heterologous expression of secondary alcohol dehydrogenase (*sadh*) in the thermophilic bacterium. *sadh*-expressing strains were combined with acetone-producing strains, to obtain an IPA-producing strain. The strain produced IPA as a major product using hexose and pentose sugars as substrates (81% mol-IPA/mol-sugar). Furthermore, IPA was produced from CO, whereas acetate was an abundant byproduct. Fermentation using syngas containing both CO and H_2_ resulted in higher IPA production at the specific rate of 0.03 h^−1^. The supply of reducing power for acetone conversion from the gaseous substrates was examined by supplementing acetone to the culture, and the continuous and rapid conversion of acetone to IPA showed a sufficient supply of NADPH for Sadh.

**Conclusions:**

The successful engineering of *M. thermoacetica* resulted in high IPA production from sugars. *M. thermoacetica* metabolism showed a high capacity for acetone conversion to IPA in the gaseous substrates, indicating acetone production as the bottleneck in IPA production for further improving the strain. This study provides a platform for IPA production via the metabolic engineering of thermophilic acetogens.

## Introduction

Petrochemistry has contributed remarkably to the development of society and has benefited our daily lives. Petroleum is one of the useful fossil resources for producing materials such as nylon and plastics; however, such resources are limited, and their consumption emits abundant greenhouse gases, including carbon dioxide (CO_2_). Therefore, seeking alternatives with carbon-neutral or -negative is imperative.

Using renewable feedstock and biomass is a globally attractive alternative. Unlike chemical reactions, biological reactions that convert biomass are energy-saving and environmentally friendly because they do not require high pressures or high temperatures. Microbial conversion possesses great potential in producing various chemicals from biomass-derived sugars [1]. Biomass-derived sugars are valuable foods and are used in several microbial applications, such as the medicinal chemicals and food additives production. Therefore, using non-sugar components of biomass for such applications is a practical approach. Non-sugar components, such as lignin, are difficult to digest and use; however, recent advancements in microbial technologies have provided another strategy for converting them via biomass gasification. Gasification is a thermochemical process that produces syngas containing mainly CO, H_2_, and CO_2_ [2, 3], enabling the use of non-sugar parts of biomass. These gaseous substrates can be converted into commodity chemicals via a microbial conversion process called gas fermentation [4]. Moreover, gas fermentation can be achieved using various resources, including waste gases such as those from steel mills, and ultimately, CO_2_ from the atmosphere.

Gas fermentation is performed with the help of acetogens [5], because they have a metabolic capacity to convert diverse carbon sources, such as sugars, organic acids, alcohols, and gaseous substrates [5, 6]. They grow autotrophically on CO_2_ and CO as carbon sources and H_2_ and CO as energy sources. As the name suggests, acetate is the primary metabolite obtained via carbon conversion. However, some acetogens produce chemicals other than acetate such as ethanol, lactate, and butanediol, which can be used for producing diverse chemical commodities. For instance, a *Clostridium autoethanogenum* strain was obtained from nature as a high ethanol producer, and its fermentation was scaled up to an industrial level [4]. Furthermore, recent advancements in genetic modifications via metabolic engineering have broadened the product spectrum [7, 8]. Target chemicals for gas fermentation are mainly small molecules that contain 2–4 carbons (C2–C4 compounds). C1 gas fermentation is similar to a C1 chemical process (C1 biotechnology) [9].

Generally, thermophilic fermentation has multiple advantages [10, 11]. Fermentation at higher temperatures can lower the risk of contamination by mesophilic microorganisms that hinder the reaction. Additionally, increasing the reaction temperature improves the overall reaction rate. Furthermore, the necessity to cool down the heat produced during fermentation can be minimized, thereby reducing the energy cost. The approach is beneficial for product recovery, which is one of the most energy-intensive steps. Moreover, when the target product is volatile, fermentation can be combined with distillation. One example is acetone, whose boiling point is 56℃. Simultaneous distillation of acetone produced during fermentation is possible because the temperature required for the growth of thermophilic microorganisms is higher than 56 ℃. The economic and technical feasibility of such a process has been evaluated, and a thermophilic acetogen, *Moorella thermoacetica*, has been successfully engineered to produce acetone from gaseous substrates [12, 13].

In the present study, we expanded the product spectrum via the thermophilic bioconversions of sugars and gaseous substrates, enabling the production of isopropanol (IPA). Owing to its higher energy density than that of acetone (acetone, 22.6 MJ/L; IPA, 23.9 MJ/L) and less corrosiveness to the engine, IPA is more desirable for fuel production [14]. Additionally, IPA is a platform chemical; thus, fewer steps are required to convert IPA into polypropylene than those required for acetone conversion. Nevertheless, separating IPA from a culture medium is challenging because the boiling point of IPA (82 ℃) is higher than that of acetone, and IPA easily forms an azeotrope in water. However, methods such as gas stripping, membrane separation, and a combination of both have been developed to isolate IPA from a culture medium. High temperatures enhance gas stripping efficiency during thermophilic bioconversion, and membrane separation can further purify the product [15, 16]. Hence, IPA production by thermophilic bioconversion is preferable in order to reduce separation costs. Therefore, herein, we engineered a thermophilic acetogen, *M. thermoacetica*, for IPA production.

## Results

### Design of IPA biosynthesis pathways in *M. thermoacetica* metabolism

**Fig. 1:**
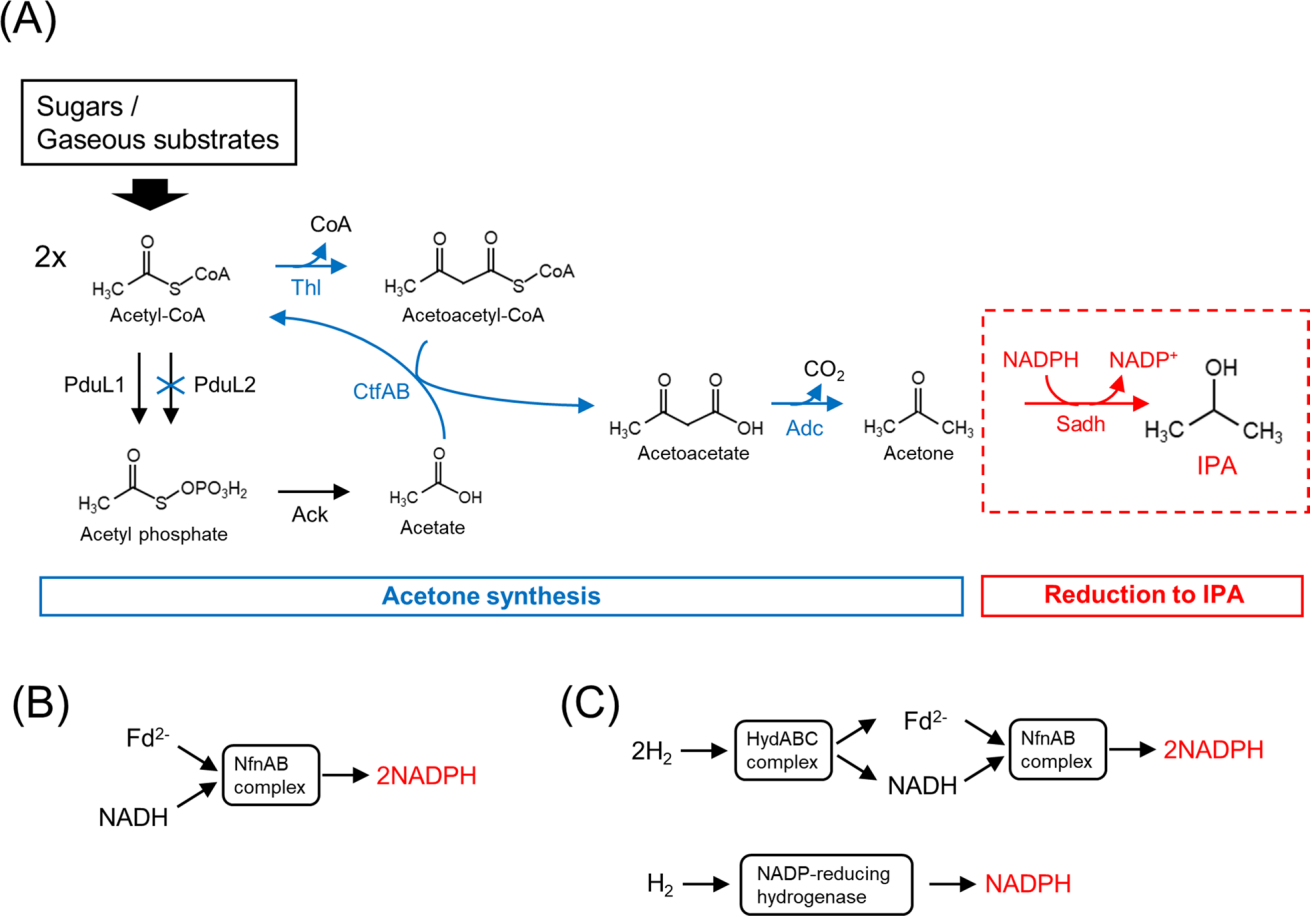
Designed IPA production pathway and NADPH supply. **A** Pathway for the synthesis of IPA from sugars and gaseous substrates. A key intermediate, acetyl-CoA, was processed by heterologously expressed enzymes for acetone synthesis (blue color) and Sadh (red color). Acetate, provided by the native pathway, was used to receive CoA from acetoacetyl-CoA. One of the genes, *pduL2*, in the acetate pathway was disrupted to enhance the carbon flow to IPA. One molecule of NADPH is required to convert acetone into IPA. Thl, thiolase; CtfAB, CoA transferase; Adc, acetoacetate decarboxylase; PduL1 and PduL2, phosphotransacetylase; Ack, acetate kinase. **B** NADPH supply using reducing equivalents derived from sugar metabolism. The sugar metabolism provided reduced form of ferredoxin (Fd^2−^) and NADH. The NfnAB complex transferred electrons from Fd^2−^ and NADH to NADP^+^, simultaneously. **C** In the presence of H_2_, NADPH was formed via two pathways. One was via Fd^2−^ and NADH. The HydABC complex transferred electrons from H_2_ to Fd and NAD^+^, followed by the transfer by NfnAB complex. The other pathway was a direct electron transfer from H_2_ to NADP^+^

IPA can be synthesized by acetone reduction in which the carbonyl group of acetone is reduced to a hydroxyl group, by the action of secondary alcohol reductase (Sadh) (Fig. 1A). Acetone can be enzymatically synthesized from two acetyl-CoA molecules, provided by catabolizing sugars or gaseous substrates in *M. thermoacetica* [6]. Acetone production by engineered *M. thermoacetica* strains has been reported [12]. The available genomic information indicates that *M. thermoacetica* does not possess Sadh homologs [17], and no IPA production by engineered *M. thermoacetica* strains indicates the same [12]. Therefore, heterologous expression of a thermophilic Sadh in the acetone-producing strain may provide IPA, given the sufficient supply of cofactors for the Sadh reaction. As a thermostable Sadh, we selected one from a thermophilic bacterium, *Thermoanaerobacter pseudoethanolicus* (formerly *Thermoanaerobacter ethanolicus*), that had been characterized to reduce acetone in an NADPH-dependent manner [18]. In *M. thermoacetica*, NADPH is supplied by electron transfers via NADH and reduced ferredoxin (Fd^2−^) or directly from H_2_ (Fig. 1B, C) [19–21].

### Heterologous expression of *sadh* in *M. thermoacetica*

**Fig. 2:**
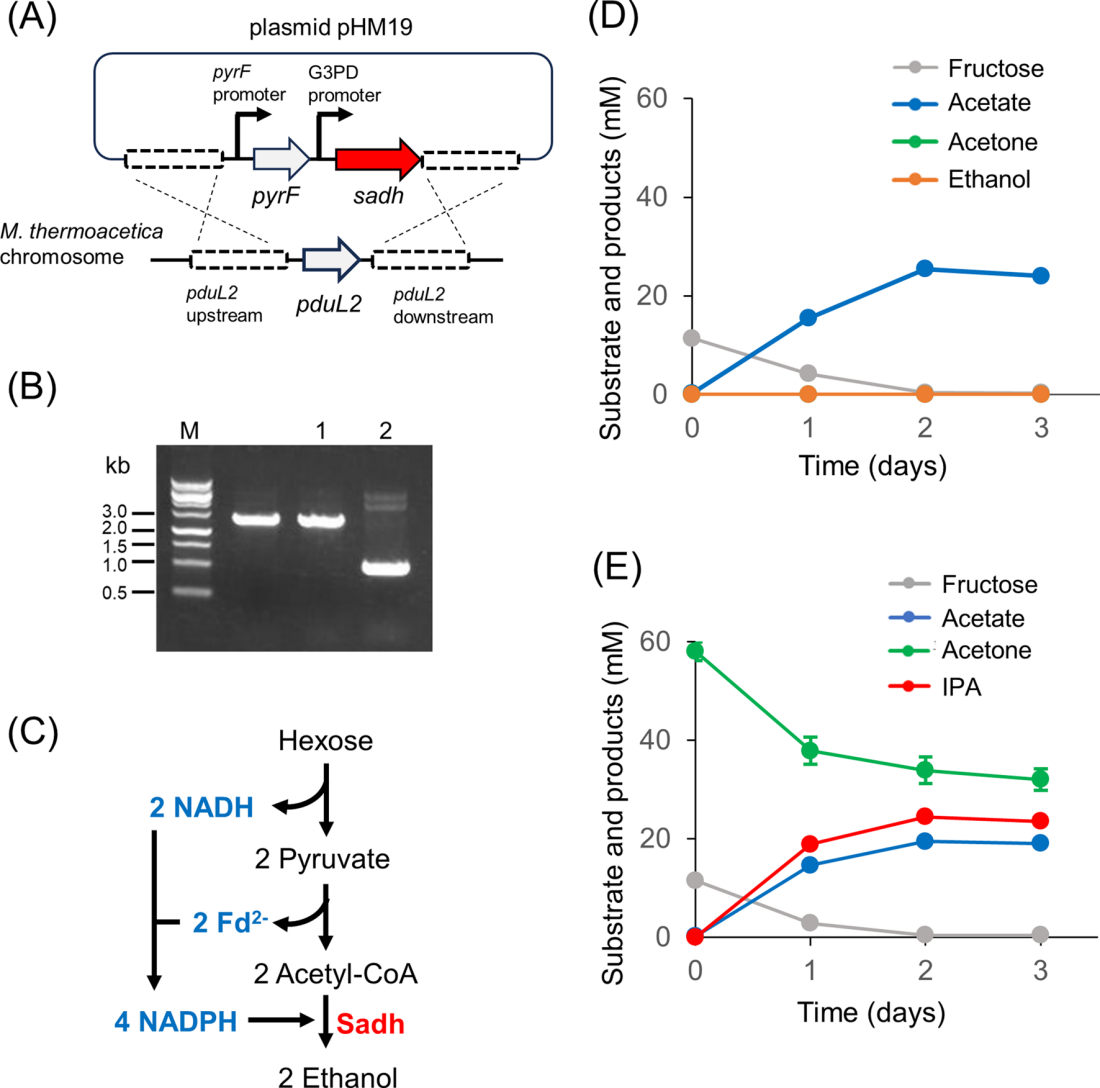
Introduction of *sadh* in *M. thermoacetica*. **A** Schematic representation of plasmid construction and *sadh* introduction in place of *pduL2*. **B** Agarose gel electrophoresis following PCR amplification of the *pduL2* region of the host and a recombinant strain. M, DNA size marker; 1, the pduL2::sadh strain; 2, the ∆*pyrF* strain. The size of the amplified region was shifted to 2.6 kb in the pduL2::sadh strain, consistent with the DNA construct, which was originally 0.9 kb in the ∆*pyrF* strain. **C** A plausible metabolic pathway showing cofactor supply for ethanol production from hexose. Reducing equivalents NADH and reduced Fd were converted into NADPH via enzymatic electron confurcation. NADPH is a cofactor for Sadh. **D** Culture profile of the pduL2::sadh strain supplemented with fructose as the substrate. **E** Acetone supplementation to the culture of the pduL2::sadh strain. The condition was the same as in **D**. Data are presented as the mean with SDs of two biological replicates in **D**, **E**. Most error bars are smaller than the symbols of data plots

Ethanol can be produced by acetyl-CoA reduction using Sadh [18]. Therefore, ethanol production should be monitored to determine whether *sadh* is functionally expressed in *M. thermoacetica*. Although ethanol is an unwanted byproduct of IPA production, this is a simple test for evaluating the functionality of *sadh* in *M. thermoacetica*. Thus, we selected the wild-type background and designed a DNA construct to disrupt *pduL2* upon *sadh* introduction. PduL2 is an enzyme responsible for acetate production, and *pduL2* disruption lowers the carbon flow to acetate, enabling the clear detection of ethanol [22]. A constitutive glycerol-3-phosphate dehydrogenase (G3PD) promoter was used to drive the expression of the codon-optimized *sadh* gene in *M. thermoacetica* (Fig. 2A). Further, the construct was successfully introduced into the *M. thermoacetica* genome using *pyrF* as a selection marker, and the pduL2::sadh strain was obtained (Fig. 2B).

Sadh catalyzed ethanol production from acetyl-CoA using two molecules of NADPH [18]. In *M. thermoacetica* grown on sugars, NADPH was produced by electron confurcation [19] (Fig. 1B), which is a reaction carried out by the NfnAB complex to obtain two NADPH molecules by simultaneously oxidizing one NADH molecule and one Fd^2−^ molecule. Two NADH molecules and two Fd^2−^ molecules are obtained by the oxidation of one fructose molecule to two acetyl-CoA molecules; thus, four molecules of NADPH should be obtained via electron confurcation. Herein, the two acetyl-CoA molecules were reduced by the four NADPH molecules, providing the redox balance for ethanol formation (Fig. 2C).

The pduL2::sadh strain was grown using fructose as a substrate, and resultant metabolites in the culture supernatant were analyzed. Because only acetate was produced from fructose, we inferred that Sadh was not functional in *M. thermoacetica* (Fig. 2D). Another possibility was that Sadh was active but did not reduce acetyl-CoA in *M. thermoacetica*; hence, we directly examined acetone reduction activity by adding acetone to the culture medium. Externally added acetone was rapidly consumed, and IPA was produced (Fig. 2E). IPA production was dependent on acetone supplementation, and the amount was stoichiometric, indicating that Sadh catalyzed acetone reduction to IPA. Moreover, significantly lower levels of acetate were produced in the acetone-supplemented culture than in the culture without acetone supplementation. When acetone was not added, 25.5 mM of acetate was produced from 11.4 mM of fructose, whereas when acetone was added, 19.5 mM of acetate and 24.5 mM of IPA were produced from 11.6 mM of fructose and 24.2 mM of acetone, respectively. The cofactor supply for IPA production explains the production of lower acetate levels (see the Discussion section).

### Construction of an IPA-producing strain of *M. thermoacetica*

**Fig. 3:**
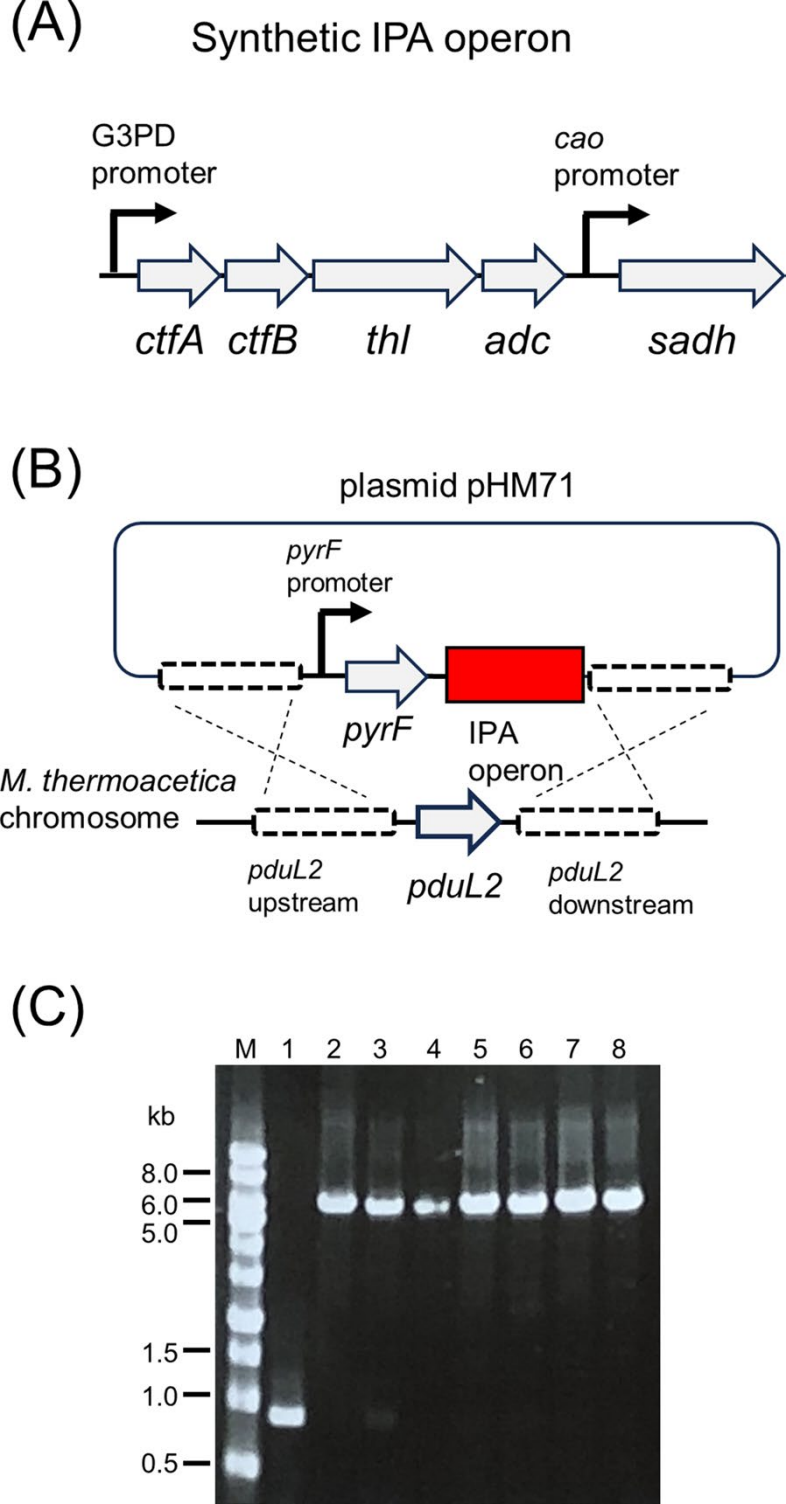
Introduction of genes for IPA production in *M. thermoacetica*. **A** Schematic representation of the synthetic IPA operon encoding enzymes for IPA production from acetyl-CoA. The reactions by enzymes derived from corresponding genes are shown in Fig. 1. **B** Schematic representation of the plasmid construction and introduction of the synthetic IPA operon in place of *pduL2*. **C** Agarose gel electrophoresis, following PCR amplification of the *pduL2* region of the host and recombinant strains. M, DNA size marker; 1, the ∆*pyrF* strain; 2, the plasmid pHM71 (positive control); 3–8, candidates of the pduL2::IPA strain. The size of the amplified region was shifted to 6.5 kb in the pduL2::IPA strain, consistent with the DNA construct, which was originally 0.9 kb in the ∆*pyrF* strain. A candidate clone (lane No. 3) showed a faint band with the same migration as the ∆*pyrF* strain, which indicated a mixed population. This clone was excluded. The other clones showed the same culture profile in the following fermentation analysis

Acetone synthesis and reduction by Sadh provide IPA for *M. thermoacetica* metabolism. We selected the acetone synthesis pathway and previously established thermophilic enzymes to construct the required strain [12]. In the designed DNA construct, *sadh* from *T. pseudoethanolicus* was located downstream of the synthetic acetone biosynthesis gene cluster (Fig. 3A). The G3PD promoter transcribed genes related to the acetone synthetic pathway. Because the G3PD promoter controls four genes responsible for acetone biosynthesis, *sadh* added downstream may not be sufficiently expressed. Therefore, we selected another constitutive promoter of the gene that encodes a copper amine oxidase-like protein (Moth_2343) to express *sadh*. The introduced IPA biosynthesis gene cluster targeted the *pduL2* locus; thus, *pduL2*, which was responsible for acetate production, was deleted to increase the carbon flow to IPA production (Fig. 3B). Although IPA biosynthesis requires acetate as an intermediate, another phosphotransacetylase activity derived from *pduL1* can supply acetate (Fig. 1A). Next, the DNA construct was introduced into *M. thermoacetica* genome using *pyrF* as a selection marker. Further, the resultant clones were subjected to repeated colony isolation on an agar medium to obtain a single genotype (Fig. 3C). Ultimately, pduL2::IPA, a strain with the IPA biosynthetic pathway was obtained.

### IPA production from hexose and pentose sugars

**Fig. 4:**
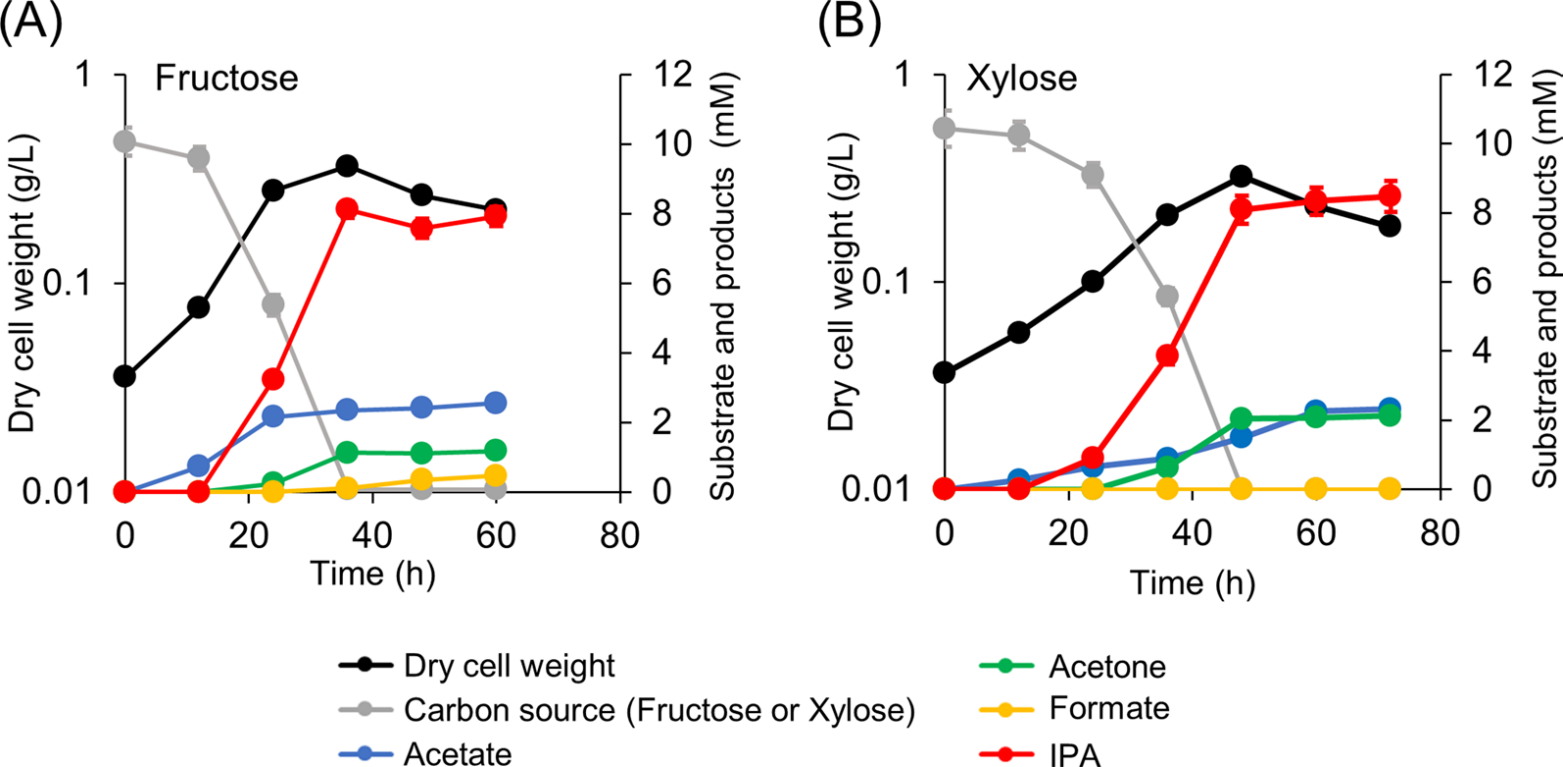
Culture profiles of the pduL2::IPA strain when supplemented with hexose (fructose) and pentose (xylose) sugars. The consumption of fructose (**A**) or xylose (**B**) was monitored and products including acetate, acetone, formate, and IPA, in the culture supernatant were measured at each time point. Dry cell weight was calculated according to the OD. Data are presented as the mean with SDs of three biological replicates. Most error bars are smaller than the symbols of data plots

The pduL2::IPA strain was then evaluated for IPA production from sugars. First, the strain was batch-cultured in a medium containing fructose as a hexose-sugar substrate. The cell growth stopped at 36 h when the supplied fructose was completely consumed (Fig. 4A). The primary product was IPA (8.1 mM of IPA from 10.1 mM of fructose) and its yield was 81% mol-IPA/mol-fructose (Table 1), which was higher than IPA produced from hexoses using engineered *Escherichia coli* [23, 24].

**Table 1:** IPA production profile from sugars

Sugar	IPA/substrate (% mol/mol)	Specific production rate for IPA (h^−1^)^a^	Carbon recovery for IPA
Fructose	81	0.076	0.40
Xylose	81	0.079	0.49

^a^g-IPA produced per g-cell per hour

Additionally, an acetone precursor was detected (1.2 mM). Further incubation after fructose consumption did not change the acetone concentration, indicating that acetone conversion to IPA stopped upon complete fructose consumption. Initially only acetate was produced at 12 h, suggesting its role as an intermediate in acetone and IPA production. Further, the acetate concentration increased to 2.6 mM at 24 h and remained almost constant throughout the IPA production. Nevertheless, fructose was still consumed after this time. A low concentration of formate (0.5 mM) was detected after complete fructose consumption. Formate is produced by CO_2_ reduction via NADPH in the first step of the Wood–Ljungdahl pathway [25].

*M. thermoacetica* uses xylose, a pentose sugar, as well as hexose sugars. Similar to fructose supplementation, xylose was supplied to the pduL2::IPA strain for IPA production (Fig. 4B). Cell growth stopped at 48 h when the supplied xylose was consumed completely, and IPA was the main product (8.5 mM IPA from 10.4 mM xylose). The IPA yield was 81% mol-IPA/mol-xylose (Table 1). Additionally, acetone (2.1 mM) and acetate (2.3 mM) were produced. Initially, only acetate was produced, indicating its role as an intermediate.

### IPA production from CO and syngas

**Fig. 5:**
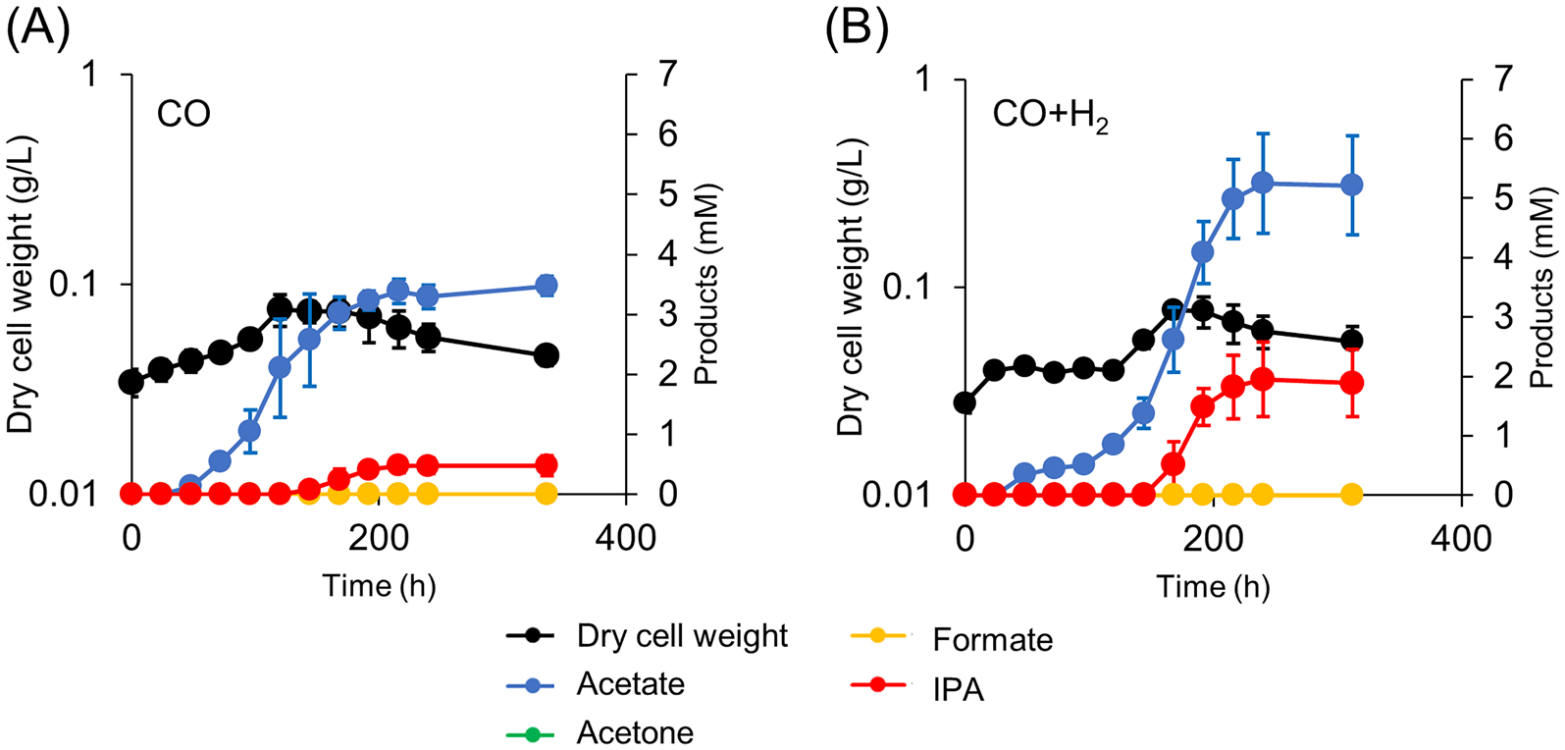
Culture profiles of the pduL2::IPA strain when supplemented with gaseous substrates, CO (**A**) or syngas (CO:H_2_ = 1:1) (**B**). Products including acetate, acetone, formate, and IPA, in the culture supernatant were measured at each time point. Dry cell weight was calculated according to the OD. Data are presented as the mean with SDs of three biological replicates

*M. thermoacetica* autotrophically grows and feeds on H_2_, CO, and CO_2_. Previously, we have reported acetone production from CO and syngas in an autotrophic manner using engineered *M. thermoacetica* [12]. The present study suggested that Sadh introduction might be responsible for IPA production from gaseous substrates. Thus, we analyzed IPA gas fermentation in a batch culture filled with gaseous substrates. First, we used only CO as the energy source (Fig. 5A), and the partial pressure of CO was maintained at 0.04 MPa in the culture bottles. The results showed that initial acetate production (3.5 mM) was followed by IPA production (0.5 mM).

Low IPA production could potentially be increased by supplying H_2_ as the energy source and reductant because acetone production was increased by H_2_ addition in a previous study [12]. We added H_2_ at a partial pressure of 0.04 MPa in addition to CO for the gas fermentation experiment (CO:H_2_ = 1:1). Consequently, IPA production increased to 2.0 mM, which was 4 times higher than that of CO (Fig. 5B). Furthermore, acetate production increased to 5.2 mM, which was 1.5 times higher than that of CO. H_2_ supplementation enhanced IPA production more significantly than acetate production, as seen in the case of acetone production. This resulted in increased IPA selectivity (Table 2). However, the specific rate of IPA production was lower than that of acetone production. Under the same gas composition, the acetone production rate was 0.09 h^−1^ (g-acetone produced per g-cell per hour), whereas that of IPA production was 0.03 h^−1^ (g-IPA produced per g-cell per hour) [12].

**Table 2:** IPA production profile from gaseous substrates

Gaseous substrate	Specific production rate for IPA (h^−1^)^a^	IPA selectivity (IPA-mol/Acetate-mol)
CO	0.006	0.14
CO + H_2_	0.031	0.36

^a^g-IPA produced per g-cell per hour

In both cases, acetate was produced initially, followed by IPA production. This is consistent with the IPA metabolic pathway (Fig. 1), in which acetate is required for acetone and IPA synthesis. No acetone was observed throughout the culture, indicating that acetone was reduced upon its synthesis.

### Acetone reduction capacity of *M. thermoacetica* using gaseous substrates

**Fig. 6:**
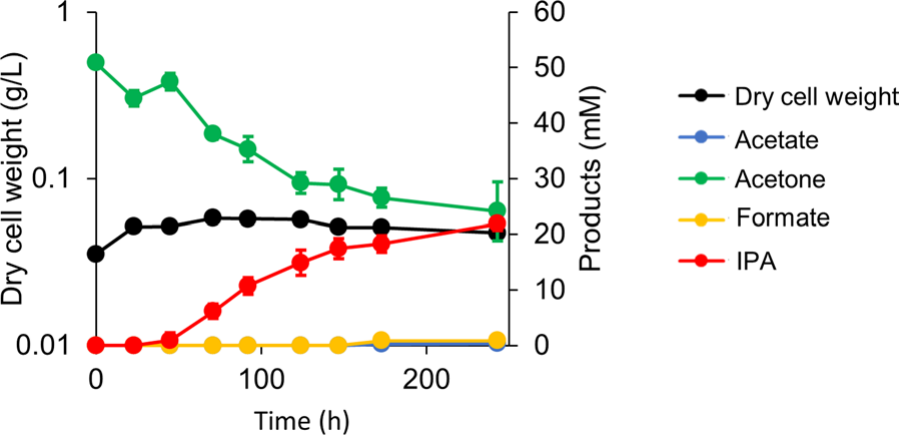
Culture profiles of the pduL2::IPA strain when supplemented with syngas for testing acetone reduction. Acetone (50 mM) was added to the culture medium in the culture vial filled with syngas (CO:H_2_ = 1:2), followed by inoculation and starting culture. Products including acetate, acetone, formate, and IPA, in the culture supernatant were measured at each time point. Dry cell weight was calculated according to the OD. Data are presented as the mean with SDs of three biological replicates. Some error bars are smaller than the symbols of data plots

Acetone was not detected during gas fermentation by the pduL2::IPA strain, which suggested that all acetone was reduced to IPA (Fig. 5). This result indicated that intracellular NADPH was sufficient to reduce all acetone produced in the cells. Generally, the supply of a reductant can be a potential bottleneck in IPA production; however, this challenge was not observed for the pduL2::IPA strain. Therefore, we estimated the supply of NADPH in the cells from gaseous substrates. To serve this purpose, acetone was extracellularly added to the culture medium and its conversion to IPA was measured. Syngas was used as the gaseous substrate, and an excess amount of acetone was added (25 times more than the amount of IPA produced in the syngas condition, which is shown in Fig. 5B). Acetone reduction was initiated 45 h after the inoculation. The reaction rate was constant up to 147 h (Fig. 6). Nearly half of the supplied acetone was consumed and a similar amount of IPA was produced. No acetate production and only slight cell growth were observed. The reducing power of syngas was preferentially used for acetone reduction, and cell growth metabolism was maintained at a minimum level under such conditions.

## Discussion

### Selectivity of Sadh in *M. thermoacetica* for reducing acetone

Previously, *M. thermoacetica* was genetically engineered to produce acetone. However, this strain did not produce IPA [12]. In the present study, the heterologous expression of *sadh* from *T. pseudoethanolicus* conferred acetone-reducing activity to *M. thermoacetica*, leading to the successful construction of a strain for IPA production. This was contrasting to the case of *Clostridium autoethanogenum*, in which a native gene encoding Sadh is present in its genome that needs to be deleted to construct acetone-producing strains to avoid IPA production [26, 27].

Conversely, a previous report showed that Sadh from *T. pseudoethanolicus* reduced acetyl-CoA to ethanol in vitro [18]. *T. pseudoethanolicus* natively produces ethanol from acetyl-CoA using multiple aldehyde and alcohol dehydrogenases. One proposed pathway for ethanol production is the two-step reduction of acetyl-CoA by Sadh using two NADPH molecules. Therefore, *sadh* expression may lead to ethanol production in *M. thermoacetica*. However, no ethanol production was observed in the *pduL2*-deleted strain, which slowed the carbon flow to acetate. The reported activity of Sadh for acetyl-CoA reduction is 5.5 U/mg [18], whereas that of PduL1, which is responsible for the remaining acetate production, is 265 U/mg [28]. The difference in the activity of these competing enzymes might explain no ethanol production. As for the IPA production pathway, the thiolase activity against acetyl-CoA is 74.4 U/mg [29]. Consistently, the pduL2::IPA strain did not produce ethanol, either. This result could explain the selective production of IPA from acetyl-CoA.

### NADPH supply from sugars for Sadh reaction

Although Sadh is functionally expressed in *M. thermoacetica*, NADPH supply is essential for its reducing activity. When the pduL2::sadh strain was analyzed for IPA production with extracellularly supplemented acetone in fructose-supplemented culture, NADPH was supplied in the form of reducing equivalents derived from fructose metabolism. When one fructose molecule was converted into two acetate molecules by glycolysis and subsequent reactions, including pyruvate oxidation, two NADH and two Fd^2−^ molecules were provided. In the absence of acetone, these reducing equivalents were used to fix two CO_2_ molecules and convert them into one acetate molecule, as observed in the wild-type strain. When acetone was supplied to the pduL2::sadh strain, the reducing equivalents were alternately used to convert acetone into IPA. During electron confurcation, electrons from two NADH and two Fd^2−^ molecules were used to form four NADPH molecules. In this context, the lowered amount of acetate produced matched the amount of IPA produced; when acetone was provided, acetate production was lowered by 6.0 mM compared to the absence of acetone (Fig. 2D, E). This was equivalent to 24.0 mM of reducing equivalents. Furthermore, 24.2 mM of acetone was reduced to IPA, which quantitatively explained the supply of reducing power.

A similar scenario would provide the reducing equivalents when IPA is produced directly from sugars and affects the theoretical yields. Herein, when acetone was produced from fructose, the redox balance of the entire pathway was the same as that for acetate production. *M. thermoacetica* performs a near stoichiometric conversion of hexose sugars to acetate [6]:

$${\text{C}}_{{6}} {\text{H}}_{{{12}}} {\text{O}}_{{6}} \to {\text{3 acetate}}$$

No reduction or oxidation reactions were present both in acetate and acetone syntheses initiated from acetyl-CoA. Therefore, in theory, the maximum acetone production is half of the acetate production:

$${\text{C}}_{{6}} {\text{H}}_{{{12}}} {\text{O}}_{{6}} \to {1}.{\text{5 acetone }} + { 1}.{\text{5 CO}}_{{2}} + { 1}.{\text{5 H}}_{{2}} {\text{O}}$$

However, IPA production requires one reducing equivalent to convert one acetone molecule into IPA. To serve this purpose, a fraction of the reducing power to fix CO_2_ [21] was used. Therefore, the maximum IPA production was as follows:

$${\text{C}}_{{6}} {\text{H}}_{{{12}}} {\text{O}}_{{6}} \to {1}.{\text{3 IPA }} + {\text{ 2 CO}}_{{2}} + \, 0.{\text{7 H}}_{{2}} {\text{O}}$$

When pentose was supplied, the reaction was as follows:

$${\text{C}}_{{5}} {\text{H}}_{{{1}0}} {\text{O}}_{{5}} \to {1}.{\text{1 IPA }} + { 1}.{\text{7 CO}}_{{2}} + \, 0.{\text{6 H}}_{{2}} {\text{O}}$$

Based on these stoichiometric equations, IPA production from fructose was 62% (= mol-produced IPA/mol-[input fructose × 1.3] × 100) of the theoretical maximum yield, whereas xylose production reached 74% (= mol-produced IPA/mol-[input xylose × 1.1] × 100).

### Features of IPA production from gaseous substrates

IPA was successfully produced from gaseous substrates including CO and syngas, and during this production, abundant acetate production was observed, which was contrary to sugar-based fermentation. The metabolic pathway for the precursor acetone was identical to that of the previously constructed strain pduL2::acetone, which showed abundant acetate production [12]. Therefore, metabolic engineering to enhance acetone synthesis is the first step towards selective IPA production.

During IPA production from gaseous substrates in the present study, no detectable amounts of acetone were produced. The reason might be that acetone is most likely to convert into IPA as soon as it is produced. In many IPA-producing microorganisms, a major bottleneck for microbe-based IPA production is NADPH supply for reducing acetone [30]. Thus, some researchers attempted to control intracellular NADPH supply for acetone reduction [24, 31]. However, acetone was completely reduced when the engineered *M. thermoacetica* produced IPA from gaseous substrates. The activity of Sadh and the NADPH pool were sufficient to reduce acetone during its synthesis from gaseous substrates. This was contradictory to IPA production from gaseous substrates using engineered *Acetobacterium woodii*, in which one-third of acetone was not reduced [32]. Further, we evaluated the abundance of NADPH by supplying an excessive amount of acetone extracellularly. Acetone was subsequently reduced, and the reducing power was preferentially consumed for acetone reduction rather than for cell growth or other metabolite production (Fig. 6). Well-characterized electron bifurcation and confurcation reactions are observed in *M. thermoacetica* for the production of NADPH from H_2_, and NADPH-dependent hydrogenase activity also serves for direct NADPH production from H_2_ [19–21]. However, the contribution of these two pathways to the NADPH supply remains unclear.

In summary, acetone productivity is a critical factor in IPA production from gaseous substrates by *M. thermoacetica* and not NADPH supply. Strategies for improving the performance of acetone-biosynthetic enzymes and balancing the carbon flow between acetate and acetone production are key factors for selective and high IPA production.

## Conclusions

IPA production from sugars and syngas using the engineered thermophile was successfully demonstrated in the present study. Further metabolic engineering to increase IPA production, especially from gaseous substrates, is necessary, and solutions have been proposed as improving acetone synthesis. The present study provides a basis for the thermophilic bioconversion of IPA, which would enable the simultaneous recovery of IPA, thereby increasing its productivity and lowering the production cost.

## Methods

### Strains and culture conditions

*M. thermoacetica* ATCC39073 and its engineered derivatives were used in this study (Table 3). The strains were cultured in serum bottles under anaerobic conditions at 55 ℃. When the gaseous substrates were provided for fermentation, the bottles were shaken at 180 rpm to dissolve the gases.

**Table 3:** Strains and plasmids used in this study

Strain or plasmid name	Description	Source or reference
Strains		
*Escherichia coli*		
HST08	Cloning host	TaKaRa
Top10	Modification host	Invitrogen
*Moorella thermoacetica*		
ATCC39073	Wild-type strain	ATCC
∆*pyrF*	*pyrF* gene was deleted	33
pduL2::sadh	*sadh* gene with G3PD promoter was introduced into the *pduL2* region	This study
pduL2::IPA	The thermophilic IPA operon was introduced into the *pduL2* region	This study
Plasmids		
pBAD33	Backbone plasmid for methylation plasmids	34
pBAD-M1281	pBAD33 carrying the Moth_1671, Moth_1672, and Moth_2281 for DNA methylation	33
pK18mob	Backbone plasmid for transformation plasmids	35
pK18-∆pduL2::*ldh*	A transformation plasmid to introduce *ldh* with G3PD promoter into the *pduL2* region	36
pHM19	*sadh* was cloned in pK18-∆pduL2::*ldh*, replacing *ldh*	This study
pK18-kan2	Backbone plasmid for pHM35 construct	37
pHM35	The promoter region of *cao* was cloned	This study
pHM36	The promoter region of *cao* and *sadh* were cloned	This study
pHM5	A transformation plasmid to introduce the thermophilic acetone operon into the *pduL2* region with *pyrF* marker	12
pHM71	A transformation plasmid to introduce the thermophilic IPA operon into the *pduL2* region with *pyrF* marker	This study

The culture medium contained 1.0 g of NH_4_Cl, 0.1 g of KCl, 0.2 g of MgSO_4_·7H_2_O, 0.8 g of NaCl, 0.1 g of KH_2_PO_4_, 0.02 g of CaCl_2_·2H_2_O, 2.0 g of NaHCO_3_, 10 mL of trace elements, 10 mL of Wolfe’s vitamin solution, and 1.0 mg of resazurin per liter of deionized water [38, 39]. The pH of the medium was adjusted to 6.9 with 6N HCl. The medium was prepared anaerobically by boiling and cooling under an N_2_/CO_2_ (80/20 ratio) atmosphere. After cooling, the medium was dispensed into 125-mL serum bottles under the N_2_/CO_2_ atmosphere. The serum bottles were crimp-sealed and autoclaved. Yeast extract and L-cysteine·H_2_O stock solutions were prepared separately and added to the medium before inoculation to reach a final concentration of 1.0 and 1.2 g/L. The total volume of the medium was adjusted to 50 mL. When sugars were supplied as substrates, either fructose or xylose stock solutions were prepared separately and added to the medium to reach 10–11 mM as the final concentration. The gaseous substrates were added to the headspace of the bottles by adjusting the partial pressure during injection.

### Plasmid construction

The plasmids were constructed using In-Fusion HD Cloning Kit (Clontech Laboratories, TaKaRa Bio, Shiga, Japan), following polymerase chain reaction (PCR) amplification of the insert and vector DNA fragments using KOD FX Neo (Toyobo Co., Ltd., Osaka, Japan). DNA primer sequences used during PCR are listed in Table 4. The constructed plasmid was cloned into *E. coli* HST08 and confirmed by Sanger sequencing.

**Table 4:** Primers used in this study

Primer	Sequence
TK03	TCGGCCTGCTTTCATGCTTG
TK04	TATGTACTCCTCCTTATATTTATTGTAACGGCAAGG
TK01	AAGGAGGAGTACATAATGATGAAAGGCTTCGCCATGC
TK05	ATGAAAGCAGGCCGATTAGGCCAGAATGAC
JK235	TCGGCCTGCTTTCATGCTTGAC
JK236	TTACTTCAGATAATCGTAGATCACTTCGG
JK237	CGATTATCTGAAGTAAGCTTGTCATCCTACATTTCACGCC
JK238	CATGAAAGCAGGCCGATTAGGCCAGAATGACGACCGGC
Cao-F	GCTTGTCATCCTACATTTCACGCC
Cao-R	CTATCTTCCCTCCTTGAG
pHM35-F	CAAGGAGGGAAGATAGAACTTCGGCCTGCTTTCATGC
pHM35-R	ATGTAGGATGACAAGCCTAAAACAATTCATCCAGTAAAATATAATATTTTATTTTCTCCC
TK10	AAGGAGGGAAGATAGATGATGAAAGGCTTCGCC
TK11	AAGCAGGCCGAAGTTTTAGGCCAGAATGACGACC
TK06	AACTTCGGCCTGCTTTCATGC
TK09	CTATCTTCCCTCCTTGAGTTTTCGCCC
1181-up-F	CGTTCAATAGGAAGACCACAG
1181-dw-R	GCAGTAAGCTGTATCGCAATG
JK48	TCCTCCCTCTTGTCATACGC
JK49	CAGTCATAGCAGTAAGCTGTATCG

The plasmid construct to introduce *sadh* in place of *pduL2* was constructed. *sadh* DNA sequence was codon-optimized for *M. thermoacetica* and synthesized (Genewiz). The insert DNA fragment containing *sadh* was amplified using the synthesized DNA as a template and TK01 and TK05 as primers. The vector DNA fragment, containing the upstream and downstream of *pduL2* and a selection marker gene, *pyrF*, was amplified using pK18-ΔpduL2::*ldh* as a template and TK03 and TK04 as primers. The two fragments were fused and cloned, resulting in pHM19.

The plasmid pHM71 for introducing genes for IPA production instead of *pduL2* was constructed. *sadh* was placed under the promoter region and the Shine–Dalgarno sequence of *cao*, a gene encoding a copper amino oxidase-like protein (gene ID: Moth_2343). A 0.6-kb fragment upstream of the start codon of *cao* (P_*cao*_) was amplified from the genomic DNA of *M. thermoacetica* using the primers Cao-F and Cao-R. The DNA fragment was cloned into a vector plasmid amplified using pK18-kan2 as a template and pHM35-F and pHM35-R as primers. The resulting pHM35 was used to clone *sadh* under P_*cao*_. *sadh* was amplified using pHM19 as a template and TK10 and TK11 as primers. The vector plasmid was prepared by the PCR amplification of pHM35 using the primers TK06 and TK09. The two fragments were fused and cloned, resulting in pHM36. Finally, P_*cao*_-*sadh* was amplified from pHM36 using the primers, JK237 and JK238, and cloned into a vector plasmid amplified from pHM5 using the primers JK235 and JK236. The two fragments were fused and cloned, and the marker gene *pyrF* and genes for IPA production were introduced in place of *pduL2*, resulting in pHM71.

### Transformation and isolation of mutants

The genetic transformation of *M. thermoacetica* was performed according to previously described protocols [12, 33] with slight modifications. A uracil auxotrophic mutant (Δ*pyrF*) was used as the host. The strain was cultured in the basal medium containing 2 g/L of fructose as the carbon source and 10 ug/mL of uracil instead of yeast extract. The cells were grown to the log phase, harvested by centrifugation, washed in the electroporation buffer (10% glycerol and 250 mM sucrose), and used for transformation. The DNA for transformation was methylated in the *E. coli* Top 10 harboring plasmid pBAD-M1281. The transformants were selected by growing them on agar medium without uracil in roll tubes. The colonies were further purified to ensure a single population by repeating colony formation in roll tubes. DNA recombination into the genomic DNA was confirmed by PCR amplification using the primers 1181-up-F and 1181-dw-R for the pduL2::sadh strain and the primers JK48 and JK49 for the pduL2::IPA strain.

### Analytical methods

To analyze the culture, 1 mL of the cell culture was sampled and directly used to measure the optical density (OD) at 600 nm. A previously determined formula, 1 g of dry cell weight/L = 0.383 OD, was used for conversion [36]. The culture supernatant was separated by centrifugation and filtered through a 0.22-μm filter to measure the amount of supplied sugars and metabolites. The sugars and metabolites were analyzed by performing high-performance liquid chromatography (HPLC; LC-2000 Plus HPLC; Jasco, Tokyo, Japan) equipped with a refractive index detector (RI-2031 Plus; Jasco), the Shodex RSpak KC-811 column (Showa Denko, Kanagawa, Japan), and the Shodex RSpak KC-G guard column (Showa Denko). The column temperature was maintained at 60 ℃. The isocratic method was used in 0.1% (*v*/*v*) phosphoric acid at a flow rate of 0.7 mL/min.

## Data Availability

All data supporting the conclusions are included in this article.
